# Letter to the Editor: Time-Out Protocol to Ensure Understanding and Implementation of the Storm of Instructions and Protocols During the COVID-19 Pandemic

**DOI:** 10.1177/1062860620925538

**Published:** 2020-05-22

**Authors:** Ayala Kobo-Greenhut, Jakob Arad, Bar Osher Revital Levi-Hevroni, Izhar Ben Shlomo

To the Editor:

During the hectic last few weeks, we in medical service teams have noticed a recurrent pattern of a storm of frequent updating of instructions, which not rarely turn previous instructions upside down. The latter leads to much learning and requires leaping from one learning behavior (LB) to another.^1-4^ LB consists of activities through which data are obtained and processed. Frequent arrival of new protocols should result in frequent leaping from one LB to another. Herein we suggest a time-out protocol (TOP) with built-in immediate questions, which forces the reader to act hypothetically under the new instructions (activity). TOPs resulted in dramatically improved safety in surgery.^5^ It uses an interactive internet form. Every instruction is followed by an immediate question, answered *no, yes*, or *need additional explanation*. Using computers, the organizer follows team members and identifies people and elements needing re-explanations or clarification, respectively.

Our protocol begins with the following highlights:

- The coronavirus is a respiratory virus.
- Infective drops adhere to surfaces. Without adhesion drops infect through the air, primarily by spray-producing procedures. Inhalation is not a spray-producer.

Actions needed by staff while donning protective gear and approaching a patient suspected of being infected:

- Objects that can impair the integrity of the protection must be removed.
- Tie robe laces to tighten around the body.
- Surgical mask well over the nose and under the chin and sealed.
- With potential aerosol producing procedures wear an N-95 mask.
- Wear a face mask, not glasses instead.
- Gloves pulled over the sleeve.
- For respiratory support verify N95 mask, non-waterproof robe, face mask, gloves, cloth overlay on the shoes.
- Do not hesitate to supervise your peers’ proper protection.

Sample questions and answers:

1. Is it possible to wear earrings and necklaces when dealing with a patient with suspected coronavirus infection?
  Yes  No  Need further explanation
2. For patient suspected of coronavirus infection, wear an N-95 mask.
  Yes  No  Need further explanation
3. My eyewear provides adequate protection for treating a patient with suspected coronavirus infection.
  Yes  No  Need further explanation
4. If you wear gloves, you should not wash your hands when treating a patient with suspected coronavirus infection
  Yes  No  Need further explanation

Mankind is facing one of the most threatening crises ever. Usually one is required to read protocols and sign that he/she read and understood them. With frequent changes, this is not enough. System managers should go the extra mile to make sure that instructions were read and understood. We suggest here a TOP for individual staff members that does not allow shallow reading (if at all) and insists on verification of understanding of the various lines of instructions. We have every reason to believe that implementation of this approach would lead to much better adherence to guidelines and protocols as we continue fighting the coronavirus pandemic.

Ayala Kobo-Greenhut, PhD *Zefat Academic College, Safed,
Israel*Jakob Arad, MD Bar Osher Revital Levi-Hevroni, MD *Joseftal Medical
Center, Eilat, Israel*Izhar Ben Shlomo, MD *Zefat Academic College, Safed, Israel*
